# Performance Characteristics of Different Anti-Double-Stranded DNA Antibody Assays in the Monitoring of Systemic Lupus Erythematosus

**DOI:** 10.1155/2017/1720902

**Published:** 2017-12-31

**Authors:** Michael Mahler, Chelsea Bentow, Tyler O'Malley, Claudia Ibarra, John Conklin, Mary Ann R. Aure, Thierry Dervieux

**Affiliations:** ^1^Inova Diagnostics Inc., San Diego, CA, USA; ^2^Exagen Diagnostics, Vista, CA, USA

## Abstract

**Objective:**

We sought to evaluate different anti-double-stranded DNA assays for their performance characteristics in monitoring disease activity fluctuations in systemic lupus erythematosus (SLE).

**Methods:**

36 active SLE patients were followed monthly. At each study visit (total *n* = 371), blood was collected and disease activity was scored using the SELENA-SLEDAI (excluding anti-dsDNA or complement components) and by a physician's global assessment (PGA). Four anti-dsDNA tests were compared. Linear mixed-effects models with random intercept and fixed slopes were used to evaluate the relationship between the longitudinal fluctuations of disease activity and anti-dsDNA titers.

**Results:**

At enrollment, positivity for QUANTA Lite and high-avidity anti-dsDNA assay was both 64% and significantly lower than anti-dsDNA positivity by QUANTA Flash (83%) and CLIFT (96%). Linear mixed-effects modeling indicated that the change in clinical SELENA-SLEDAI scores was associated with the titers of all anti-dsDNA with QUANTA Flash yielding the highest marginal *R*^2^ (0.15; *p* < 0.01). QUANTA Flash was the only anti-dsDNA assay significantly associated with the change in PGA (marginal *R*^2^ = 0.05; *p* < 0.01).

**Conclusion:**

These data indicate that anti-dsDNA antibodies determined by QUANTA Flash have a value in monitoring SLE disease activity.

## 1. Introduction

A variety of assays on many platforms have been developed over the years to detect antibodies to double-stranded (ds) DNA, a key diagnostic marker of systemic lupus erythematosus (SLE). These assays include the Farr assay [1], the *Crithidia luciliae* indirect immunofluorescence test (CLIFT) [2], and a variety of solid-phase immunoassays [3]. As current solid-phase immunoassays have variable performances due to lack of standardization [3], CLIFT is often regarded as a reference method, owing to its high clinical specificity and the omission of radioactive labeling in contrast to the Farr assay. Recently, a novel assay that uses synthetic DNA has been developed on the BIO-FLASH system, a chemiluminescence immunoassay analyzer, and has been found to demonstrate strong association with disease activity and lupus nephritis [4–6].

Currently, rheumatologists mostly rely on disease activity scores based on organ involvement and clinical parameters. Such scores include the British Isles Lupus Activity Group (BILAG) index, the Systemic Lupus Erythematosus Disease Activity Index (SLEDAI), the Systemic Lupus Activity Measure-Revised (SLAM-R), the European Consensus Lupus Activity Measurement (ECLAM), and the Lupus Activity Index (LAI) [7]. All of the abovementioned scores have a subjective component which represents a significant drawback. Consequently, an objective and reliable variable to define disease activity in SLE patients would be of utmost utility in the clinical management of SLE patients. We previously established that clinical improvements in SLE paralleled the reduction in the titers of anti-dsDNA as determined using solid-phase immunoassays [8].

The goal of our study was to evaluate the performance of different assays for the detection of anti-dsDNA antibodies in a well-characterized cohort of SLE patients in a longitudinal study design with special focus on the assessment of disease activity.

## 2. Methods

### 2.1. Specimens

The patient cohort has been described in details in a previous report [8]. Briefly, 36 consented adult SLE patients presenting with active disease and activation of a complement system were enrolled and followed monthly. At each visit, blood was collected and plasma was isolated. Disease activity was determined monthly using the Safety of Estrogens in Lupus Erythematosus National Assessment- (SELENA-) SLEDAI [9] without anti-dsDNA and low-complement components and defined by the clinical SELENA-SLEDAI. Also, the physician's global assessment (PGA) of a disease activity visual analogue scale (0–3 points) was collected. For a total of 371 consecutive study visits of 36 patients, plasma and clinical data was available and included in the study.

### 2.2. Anti-dsDNA Antibody Assays

All specimens were tested using 4 different anti-dsDNA kits (as per the manufacturer's instructions). These consisted of the QUANTA Lite (QL) anti-dsDNA, NOVA Lite (NL) dsDNA *Crithidia luciliae* with DAPI (NL CLIFT) [10], QUANTA Flash (QF) dsDNA [4], and high-avidity (HA) anti-dsDNA (all Inova Diagnostics, San Diego, CA). All technologists were blinded to the operator assessing the disease activity.

### 2.3. Statistical Analysis

Linear mixed-effects models with random intercept (subject was the random factor) and fixed slopes were used to evaluate the relationship between the longitudinal fluctuation of anti-dsDNA and the change in disease activity. In this model, the dependent variable was the clinical SELENA-SLEDAI and the independent variable was anti-dsDNA titers. Anti-dsDNA titers were log-normalized before analysis. The Mann–Whitney test was used for group comparison.

## 3. Results

**Figure 1:**
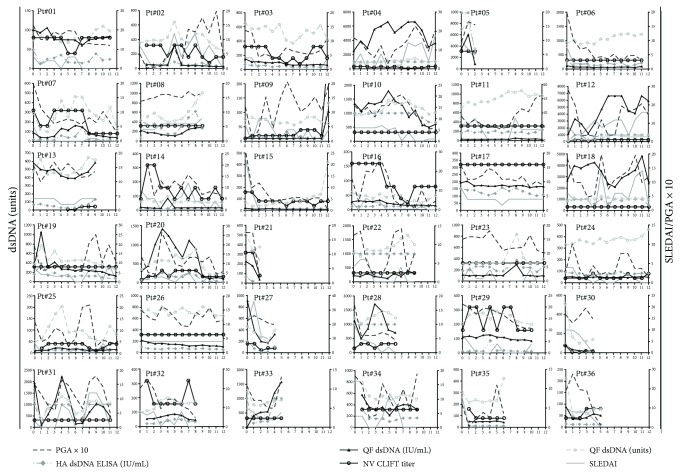
Anti-dsDNA titers and disease activity in all individual patients. Anti-dsDNA antibody titers measured at each monthly study visit are presented along with the clinical SELENA-SLEDAI and physician's global assessment (PGA × 10) at each visit.

**Figure 2:**
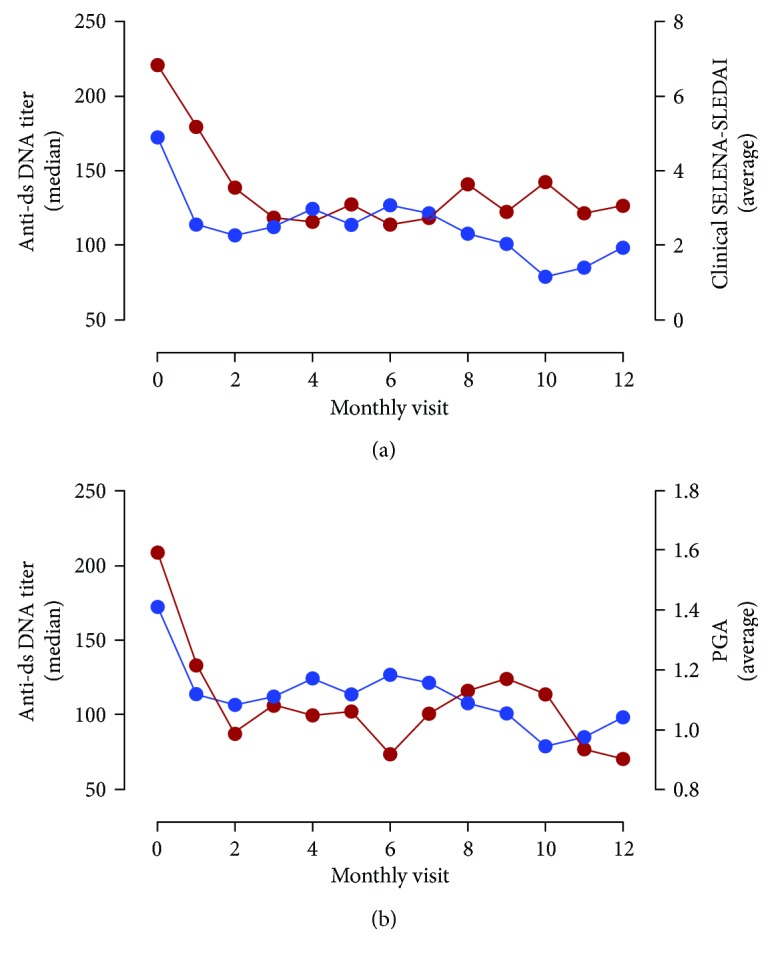
Anti-dsDNA titers (QUANTA Flash) and disease activity. Median anti-dsDNA titers (QUANTA Flash) measured at each study visit and average clinical SELENA-SLEDAI (a) and physician's global assessment (PGA) (b) are presented for each monthly study visit.

Anti-dsDNA positivity at baseline was 64% for QL (median titers: 419 units, IQ range: 208–728 units), 64% for HA (median titers: 127 units, IQ range: 32–626 units), 96% for NL CLIFT, and 83% for QF (median titers: 172 units, IQ range: 64–474 units). Baseline mean (SEM) clinical SELENA-SLEDAI and PGA scores were 6.8 ± 0.8 and 1.6 ± 0.1, respectively. Linear mixed models indicated that the fluctuations in clinical SELENA-SLEDAI were associated with QF and HA anti-dsDNA titers (*p* < 0.05) (Table 1). NL CLIFT and QL titers were not associated with the change in disease activity (*p* > 0.05). QF yielded the highest marginal *R*^2^ (0.149) as compared to other anti-dsDNA tests, thereby indicating a greater utility in tracking disease improvements. As presented in Table 1, the QF was the only assay associated with the change in PGA (*p* < 0.01; marginal *R*^2^ = 0.053).  Figure 1 illustrates the association between the change in anti-dsDNA titers by all assays and clinical improvements by clinical SELENA-SLEDAI and PGA, respectively.  Figure 2 displays the median anti-dsDNA levels derived from QF in relation to the average SELENA-SLEDAI and PGA. Study visits presenting with clinical SELENA-SLEDAI of zero point (inactive disease) presented significantly lower anti-dsDNA titers than study visits presenting with clinical SELENA-SLEDAI greater than zero point.

## 4. Discussion

Anti-dsDNA antibodies represent an important tool as aid in the diagnosis of SLE and are part of the classification criteria [11, 12]. In addition, depending on the assay used, anti-dsDNA antibody measurement can help in the assessment of DA in SLE patients [13, 14]. This is of high importance since the assessment of disease activity can be challenging for the management of SLE patients as well as for clinical trials of new drugs and it is crucial for clinicians to differentiate lupus flares from infections. Several DA scores have been established and validated that all aim at the assessment of disease activity in SLE patients. Those scores include the Systemic Lupus Activity Measure (SLAM), Systemic Lupus Erythematosus Disease Activity Index (SLEDAI), Lupus Activity Index (LAI), British Isles Lupus Assessment Group (BILAG) index, and European Consensus Lupus Activity Measure (ECLAM). In addition, several variations of the individual measures have been constructed and applied in various studies, all of them barring their individual advantages and disadvantages [15]. In our study, we employed the SELENA-SLEDAI which has been validated in several studies and is commonly used in the academic setting and also as part of outcome measure in a pivotal registration clinical trial in SLE. With the availability of new drugs for SLE, such as belimumab, flare detection and even prediction will become increasingly important. However, anti-dsDNA antibody assays are among the least standardized assays in the spectrum of autoantibody testing making their interpretation often challenging [5]. Consequently, this study aimed to compare different anti-dsDNA antibody assays as monitoring tools for SLE activity. In our analyses, CLIFT titers showed the lowest performance followed by QL and HA dsDNA. Anti-dsDNA antibodies measured by QF showed the best performance for assessing DA. This finding is consistent with that of a recent study investigating the association with DA in SLE patients presenting with lupus nephritis [4]. In this large international multicenter study of 834 SLE patients, the QF dsDNA assay showed the strongest quantitative correlation with SLEDAI-2k. The underlying reason for the enhanced performance of this novel assay for the assessment of DA in SLE patients is unclear but might be based on the technological differences with most available dsDNA assays. Differences in assay performance have been associated with different antigen sources (purity), detection methods (e.g., which immunoglobulin isotype is detected), and assay conditions (e.g., washing stringency) [16]. Historically, the majority of solid-phase assays utilized dsDNA purified from native sources which were often contaminated with DNA-binding proteins and were prone to single-strand DNA [17]. In contrast, the CLIFT method was reported to contain pure and primarily dsDNA. Based on synthetic dsDNA coupled to paramagnetic particles [5], this assay might focus on high-affinity anti-dsDNA antibodies that are associated with DA in SLE patients. One specific feature of this cohort is the high prevalence of CLIFT-positive samples which contrasts with the known moderate sensitivity of CLIFT (combined with the very high specificity). This can be explained by the high disease activity among participants in this study as prescribed in the inclusion criteria. In addition, it is important to emphasize that most studies on anti-dsDNA antibodies are based on a cross-sectional study design which has significant limitations. Longitudinal studies, such as ours, are more reliable in assessing the clinical utility for DA monitoring. It is important to point out that not all SLE patients express anti-dsDNA antibodies during the course of the disease, and therefore, other antibodies or biomarkers (such as anti-C1q, anti-chromatin, and anti-Sm) might be required to improve the serological assessment of DA in all SLE patients [17]. Taken together, the QUANTA Flash dsDNA has the potential to become the assay of choice in the monitoring of SLE disease activity.

## Figures and Tables

**Table 1:** Linear mixed-effects model of anti-dsDNA with the clinical SELENA-SLEDAI and physician's global assessment (PGA). Slope estimates (SEM) with *p* value and marginal *R*^2^ are given for each of the anti-dsDNA tested. For example, a one-log decrease in QUANTA Flash anti-dsDNA was associated with a 1.08-unit decrease in clinical SELENA-SLEDAI.

Assay	Clinical SELENA-SLEDAI	PGA
QF dsDNA	1.08 ± 0.24 *p* < 0.001 (*R*^2^ = 0.149)	0.08 ± 0.03 *p* = 0.008 (*R*^2^ = 0.053)
HA anti-dsDNA	0.77 ± 0.28 *p* = 0.006 (*R*^2^ = 0.065)	0.03 ± 0.03 *p* = 0.17 (*R*^2^ = 0.007)
QL anti-dsDNA	0.69 ± 0.4 *p* = 0.094 (*R*^2^ = 0.023)	−0.05 ± 0.05 *p* = 0.34 (*R*^2^ = 0.005)
CLIFT anti-dsDNA	0.49 ± 0.3 *p* = 0.21 (*R*^2^ = 0.008)	0.07 ± 0.05 *p* = 0.17 (*R*^2^ = 0.010)

QF: QUANTA Flash; HA: high affinity; QL: QUANTA Lite; CLIFT: *Crithidia luciliae* indirect immunofluorescence test.
